# Supplementary material for: Introducing HDAC-Targeting Radiopharmaceuticals for Glioblastoma Imaging and Therapy
Source: Pharmaceuticals (Basel). 2023 Feb 1;16(2):227. doi: 10.3390/ph16020227 (PMC9967489; doi:10.3390/ph16020227)
Supplement: Supplementary file 1 [file pharmaceuticals-16-00227-s001.zip › pharmaceuticals-2132210-supplementary.pdf]

Table S1. Representative summary of the preclinical development for candidate HDACi-derived glioma therapy.

	Regimen	Year	Main outcome and findings	Ref
Vorinostat (SAHA, Zolinza, MK0683) *Pan-HDACi*	/	2005	Growth inhibition. Single intra-tumoral injection doubled the survival time of a rat orthotopic glioma model	[1]
	/	2007	Local delivery inhibited intracranial growth in vitro and in vivo	[2]
	/	2007	Inhibited GB growth in vitro and in vivo. Suggested BBB crossing	[3]
	VAN	2009	Treatment enhanced the antiproliferative effect of VAN by inhibition of MAPK, Akt, and other downstream effectors	[4]
	/	2010	Affected glioma cell proliferation, migration, and invasion (2D and 3D)	[5]
	/	2011	Caused GSC disruption and decreased expression of EZH2 and CD133	[6]
	/	2011	Cell cycle protein modulation, activation of the G_2/M checkpoint and induction of apoptosis	[7]
	LSD1 inhibitor	2011	LSD1 and HDACs cooperate to regulate key apoptotic pathways in GB	[8]
	/	2013	Tumor growth slowdown and autophagy in GB stem cells	[9]
	Bortezomib	2013	Combination therapy significantly enhanced apoptosis	[10]
	Obatoclax/RT	2014	Bcl-2 inhibitor obatoclax overcomes resistance and radiosensitizes	[11]
	/	2014	Causes glioma cells to enter into mitosis before DNA damage could be repaired; formation of an aberrant mitotic spindle that results in glioma cell death through mitotic catastrophe-induced apoptosis	[12]
	HIRT	2015	Suggests a vital role of HDACs in the DDR and support the role of SAHA for GB treatment in combination with heavy ion therapy	[13]
	RT	2015	HDACi treatment 24 and 48-hours pre-RT caused best efficacy. Responses associated with pChek2 and Bcl-XL	[14]
	OLA	2016	Combination treatment enhanced inhibition of GB survival, induces apoptosis and impairs cell cycle progression	[15]
	/	2017	Suppressed hypoxia signaling by modulating nuclear translocation of HIF-1α	[16]
	Chloroquine	2018 2019	Autophagy inhibition potentiates SAHA-mediated apoptosis in GB cells	[17,18]
	MC1568	2019	Decreased tube formation of U87MG cells and patient-derived GB CSCs	[19]
	4HPR	2020	Combination treatment significantly reduced cell viability of rat C6 and human T98G GB cells	[20]
	/	2021	Decreased EB1 expression in GB cells, affected microtubule dynamics, cell survival and migration	[21]
	/	2022	Impairing GB cell viability, proliferation, cell motility and migration	[22]
EDO-S101* *Pan HDACi*	/	2018	Promising therapeutic activity against GB causing prolonged survival	[23]
Romidepsin (Istodax, FK228, FR901228, depsipeptide) *HDAC class I*	/	2004	Inhibited glioma cell proliferation and apoptosis was induced. In vivo, intracranial growth of transplanted GB m3-cells was inhibited	[24]
	Gamitrinib (TRAP1)	2020	Synergistic growth reduction of PDX GB cells	[25]
	Imipridones	2022	Induction of synthetic lethality and increased survival of a GB PDX model	[26]
Belinostat (PXD101, Beleodaq) *Pan-HDACi*	/	2016	Variable responses in different GB cell lines. Up-regulation of p21 mRNA expression	[27]
	/	2019	Treatment reduced tumor volume in an orthotopic rat glioma model	[28]
Panobinostat (LBH589) *HDAC class I/II*	RT	2015	Sensitized 45% of cultures. HDACi treatment 24/48-hours pre-RT resulted in the best efficacy. Responses associated with pChek2 and Bcl-XL	[14]
	Obatoclax/RT	2014	Bcl-2 inhibitor obatoclax overcomes resistance and radiosensitizes	[11]
	Bortezomib	2008	Synergistic apoptosis mediated by mitochondrial Bax translocation	[29]
	Oncolytic Virus Delta24-RGD	2015	Synergism in 50% of GSC. Toxicity to human astrocytes remained limited	[30]
	/	2017	CED of loaded pluronic nano-micelles prolongs survival in a GB rat model	[31]
	/	2017	Inhibits GB growth and angiogenesis through suppression of HIF-1α	[32]
	Bromodomain inhibitor	2018	Synergistical efficacy: inhibited cell viability and induced apoptosis of GB cells	[33]
	OTX015/Sorafenib	2018	Triple combination therapy caused significantly extended host survival	[34]
	BEZ235	2019	Synergistically inhibited cell viability; markedly inhibited cell proliferation and induced apoptosis	[35]

	Gamitrinib (TRAP1)	2020	Combination induced synthetic lethality in PDX GB cells	[25]
	Etomoxir	2020	Combination treatment demonstrated significantly prolonged survival of an PDX GB mice model	[36]
	DZ-Nep /TMZ	2020	Highest synergistic combination: DZ-Nep + panobinostat	[37]
	DZ-Nep/APR-246/ TMZ	2021	APR-246 acts in an additive manner, reducing clonogenicity and inducing apoptosis in GB cells independently of p53 status	[38]
	TMZ/LW[#]	2021	Triple combination therapy showed a cytotoxic effect against glioma cells (no immunogenic cell death seen)	[39]
	LY500307	2021	Combined treatment reduced cell viability, invasion, colony formation, enhanced apoptosis and enhanced survival of tumor-bearing mice. Could overcome the suppression of ERβ expression	[40]
	Imipridones	2022	Induction of synthetic lethality and increased survival of a GB PDX mice model	[26]
Valproic acid (VPA, valproate, Depakene) *HDAC class I*	/	1998	Restricted proliferation in the mid-G1 phase of the cell cycle and altered the prevalence and/or glycosylation state of cell surface glycoproteins	[41]
	/	1998	Inhibited proliferation and changed expression of CD44 and CD56 of malignant glioma cells in vitro	[42]
	RT	2005	Enhanced radiosensitivity of SF539 / U251 cell lines and U251 mice xenograft	[43]
	Hydrazaline[&]	2006	VPA monotherapy led to 80% growth inhibition in D54 glioma cells, potentiated by hydralazine	[44]
	Etoposide	2007	VPA sensitized glioma cells to etoposide (induced differentiation and up-regulation of p21/WAF1 expression and both isoforms of topoisomerase-II)	[45]
	/	2010	Induced cell growth inhibition and apoptotic activity in U87MG GB cells. Decreased MMP2 and MMP9 activity and enhanced expression of TIMP1	[46]
	/	2014	Caused glioma cell entry into mitosis before DNA damage could be repaired, formation of an aberrant mitotic spindle that resulted in glioma cell death through mitotic catastrophe-induced apoptosis	[12]
	RT	2015	VPA sensitized 40% of cultures. Incubation 24 and 48-hours pre-RT resulted in the best efficacy	[14]
	/	2015	Significantly reduced the proliferation rate and expression of the stem cell markers of GB-derived stem cells, indicating differentiation of the cells	[47]
	/	2016	Promoted apoptosis and inhibited Glycogen Synthase Kinase-3β through ERK/Akt signaling	[48]
	Fluvastatin	2017	Synergistic apoptosis induction	[49]
	/	2017	Inhibited GB cell growth via paraoxonase 2 expression	[50]
	Sulfasalazine	2018	Combination therapy: substantial effect on GB cell's death related to an intracellular oxidative response imbalance	[51]
	/	2018	Inhibited proliferation and reduced invasiveness in GSC through Wnt/β-catenin signalling activation	[52]
	TMZ	2019	VPA induced amphiregulin secretion confers resistance to TMZ	[53]
	/	2019	VPA increased the expression levels of acetylated histones H3 and H4 in vitro (could not be confirmed in clinical tumor samples of GB patients)	[54]
	/	2022	Induced cell apoptosis through extrinsic, intrinsic, and JAK/STAT pathways	[55]
AN-446 *VPA prodrug*	/	2018	More potent than VPA. Superior in inducing DNA damage in cancer cells, while in normal astrocytes and cardiomyoblasts AN446 was the least toxic	[56]
Trichostatin A (TSA) *HDAC 7,8*	/	2001	Induced apoptosis through an increase in Bad protein in human glioma cells	[57]
	/	2005	Inhibition of cell growth, cell cycle arrest and apoptosis induction	[58]
	2DDG	2006	Combined therapy induced strong apoptosis in brain cancer cells (non p53-dependent)	[59]
	/	2008	Induced apoptosis and cell type-specific differentiation	[60]
	MG132	2008	Synergism of apoptosis induction in U251 glioma cells upon combined treatment	[29]
	/	2009	NECL1 is a tumor suppressor. Loss of it may be caused by histone deacetylation (which can be counteracted by TSA)	[61]
	/	2010	Expression of DKK1, SFRP1 and WIF1 (potent inhibitors of Wnt signal transduction pathways) are decreased in GB, but was restored by TSA	[62]
	/	2011	TSA activates the p38MAPK-p53 cascade, which leads to Bax expression, decreased survival in C6 glioma cells	[63]
	/	2013	Promotes apoptosis, as well as augments anti-GB innate immune responses. In vivo, tumor growth of GB mice xenografts was delayed	[64]

	/	2014	TSA can inhibit proliferation, survival, tumor sphere formation, and promote differentiation of U87MG GB cells	[65]
	/	2015	Significantly reduced proliferation rate and expression of the stem cell markers of GB-derived stem cells, indicating differentiation of the cells	[47]
	CCNU	2017	Kills GB cells more efficiently than either of its monotherapies	[66]
	/	2019	Decreased tube formation of U87MG and patient-derived GB CSCs	[19]
		2022	Impairing GB cell viability and proliferation, cell motility/migration	[22]
	DZNep/BIX01294	2022	In combinations they exhibited a synergistic effect on U87MG GB cells	[67]
Entinostat (MS275) *Class I and IV*	RT	2004	Increased radiosensitivity (γH2AX foci)	[68]
	/	2006	Reduced growth of glioma cell lines, mediated by cell cycle arrest and apoptosis. In vivo, propensity to pass the BBB	[69]
	/	2009	Induced GB-derived stem-like cells to differentiate, become apoptotic and not grow as neurospheres / initiate tumor xenografts	[70]
	DOX	2011	Chemotherapy--induced apoptosis increased	[71]
	RT	2015	Radiosensitization	[14]
	/	2019	Decreased tube formation of U87MG and patient-derived GB CSCs	[19]
	MEKi	2022	Combination: more effective at reducing the GB stem-like cell markers	[72]
MC1568 *Class IIa*	/	2019	Decreased tube formation of U87MG and patient-derived GB CSCs	[19]
MPT0B291 *HDAC 6,2*	/	2020	Reduced cell viability, increased cell death and G1-phase cell cycle arrest. In vivo, therapy reduced tumor volume	[73]
	/	2021	More effective in blocking homologous recombination repair in GB cells. Reduced the growth of both TMZ-sensitive and resistant cells and prolonged survival in GB mouse model	[74]
Quisinostat (JNJ-26481585) *HDAC 1,6,9*	/	2014	Most consistent in vivo activity signals observed in GB and T-cell acute lymphoblastic leukemia xenografts	[75]
	AuNP	2021	Injectable hydrogel: successfully inhibited in vivo tumor growth	[76]
CKD5 *pan HDACi*	/	2017	Cytotoxic effects > SAHA and TSA, induced apoptosis, anti-proliferative activity and cell cycle arrest at G2/M-phase. Reduced tumor volume and prolonged the survival in vivo > TSA	[77]
Sahaquine *HDAC6*	TMZ	2018	Reduces the viability and invasiveness of GB tumoroids, as well as brain tumor stem cells. These effects are augmented upon combined therapy	[78]
Abexinostat (PCI-24781, CRA-024781) *HDAC 1,2*	LSD1 inhibitor	2011	LSD1 and HDACs cooperate to regulate key apoptotic pathways in GB	[8]
	/	2014	Attenuated cell proliferation / increased apoptosis by down-regulating EZH2, which promoted c-myc-driven apoptosis by suppressing PI3K/Akt/mTOR	[79]
	TMZ	2021	Works synergistically with TMZ	[80]
4-phenylbutyrate (4-PBA) *pan HDACi*	/	2004	4-BPA results in inhibition of cell growth. Modulates glial fibrillary acidic protein and connexin 43 expression, enhances gap-junction communication	[81]
	/	2008	GB cell lines: induced apoptosis in a dose- and time-dependent manner. All cell lines displayed unique phenotypic responses and differentiation patterns	[60]
	GEF/VAN	2011	Combined: enhanced cell killing and reduced clonogenic survival	[82]
	/	2016	Inhibited cell growth and proliferation	[83]
Sodium Butyrate (SB)	/	2001	Intratumoral infusion prolonged the survival of rats with intracerebral C6 tumors without detectable toxicity	[84]
	/	2001	Induce apoptosis through an increase in Bad protein in human glioma cells	[57]
	2DDG	2008	Combination therapy: apoptosis in brain cancer cells (p53-independent). HDACi upregulate p21, which is blocked by concomitant 2DDG	[59]
	/	2018	Induced senescence and inhibits the invasiveness of GB cells	[85]
	Quercetin	2019	Inhibited protective autophagy to enhance apoptosis in GB cells	[86]
	Curcuminoids	2021	Synergistically reduced the viability of GB cells inducing apoptosis and cell cycle arrest. Restored Wnt/β-catenin pathway antagonists gene expression	[87]
	2-DG analogs	2021	Synergistic cytotoxic effects in GB U87MG and U-251 cells	[88]
Tubastatin A *HDAC6*	Celecoxib	2017	Synergistic antitumor effects in CAL 27 and SACC-83 cells, mediated by activating the PTEN/AKT signaling pathway	[89]
	/	2019	Increased levels of DNA mismatch repair proteins MSH2 and MSH6 in TMZ-resistant GB cells. Decreased MGMT expression in TMZ-resistant GB cells	[90]
	TMZ	2019	Enhanced TMZ-induced apoptosis + reversed malignant phenotype of GB cells	[91]
	/	2020	Higher target specificity and antitumor activity compared to vorinostat	[92]
Tubacin *HDAC6*	/	2018	Suppressed glioma cell growth and drug resistance by autophagic suppression	[93]
Dacinostat	MG132%	2008	Combined: synergism of apoptosis induction in U251 glioma cells	[29]

(LAQ824)	2DDG	2008	Combined: induced strong apoptosis in brain cancer cells in a p53-independent manner	[59]
Ricolinostat (ACY-1215) *HDAC6*	/	2015	U87MG cell growth was significantly inhibited. HDAC6 increased GB growth through attenuating transforming growth factor β (TGFβ) receptor signaling	[94]
	/	2019	Increased levels of DNA mismatch repair proteins MSH2 and MSH6 in TMZ-resistant GB cells. Decreased MGMT expression in TMZ-resistant GB cells	[90]
	/	2020	Decreased JNK phosphorylation, preceding its suppression of glioma cell growth, resulting from suppression of MAPK kinase 7	[95]
CAY10603 *HDAC6*	/	2020	MAPK kinase 7 expression and JNK/c-Jun activities were suppressed in U87MG xenograft mice	[95]
	/	2019	Increased levels of DNA mismatch repair proteins MSH2 and MSH6 in TMZ-resistant GB cells. Decreased MGMT expression in TMZ-resistant GB cells	[90]
CUDC101 *pan HDACi*	Erlotinib	2016	Inhibited proliferation of erlotinib-resistant GB cells, partially restored their sensitivity to erlotinib	[96]
A452 *HDAC6*	/	2019	Increased levels of DNA mismatch repair proteins MSH2 and MSH6 in TMZ-resistant GB cells. Decreased MGMT expression in TMZ-resistant GB cells	[90]
MC2129 HDAC Class I/IIb	/	2019	Stronger cytotoxic and antiproliferative effect compared to SAHA (cell cycle arrest in the G2/M-phase)	[97]
Mocetinostat (MGCD0103) *HDAC1/2*	/			
MC1746 HDAC Class I/IIb	/	2019	Antiproliferative effects shown in U87MG glioma cells	[97]
Scriptaid	OVT	2015	Synergism in 50% of the patient-derived GSC	[30]
	RT	2015	Sensitized 40% of the cultures. Incubation 24 an 48-hours pre-RT resulted in the best efficacy of combination treatment	[14]
	/	2010	Induction of apoptosis and reduction of glioma cell proliferation through JNK activation and reduction of telomerase activity	[98]
PCl34051 *HDAC8*	/	2008	No cytotoxicity to U87MG glioma cells. IC50 = 17 μM	[99]
Givinostat (ITF2357) *pan HDACi*	/	2019	Reverted transformed phenotype and counteracted stemness in GB (in vitro and in vivo). Efficiently passed the BBB in mice	[100]
	Autophagy inhibitor	2016	Reduced viability and self-renewal ability of GSC cultures but not in differentiated GB cells and normal mesenchymal human stem cells	[101]
	/	2022	Liposomes: inhibited human GB cell growth in 2D and 3D models	[102]
RGFP109 *HDAC 1,3*	TMZ	2016	Overcomes TMZ resistance by blocking NF-κB-dependent transcription in GB cells	[103]

*/ = monotherapy. 2DDG = 2-deoxy-d-glucose, 4HPR = 4-BPA = 4-phenylbutyrate; N-(4-hydroxyphenyl) retinamide); AuNP = gold nanoparticles; BBB = Blood-Brain Barrier; BIX01294 = inhibitor of histone methyltransferase G9a; CCNU = lomustine; DSB = Double-stranded DNA break; CSCs = cancer stem cells; DDR = DNA Damage Response; DOX = Doxorubicin; DZNep = inhibitor of lysine methyltransferase EZH2 (3-deazaneplanocin A); GB = glioblastoma; CED = convection enhanced delivery; GEF = Gefinitib; GSC = glioma stem cell; HDAC(i) = Histone deacetylase (inhibitor); HIRT = Heavy ion radiotherapy; JAK/STAT = Janus kinase signal transducer and activator of transcription pathways; JNK = Jun N-terminal kinase; LDH = Lactate dehydrogenase; LW = Lophophora williamsii extract; MAPK = Mitogen-activated protein kinase; MEKi = MAPK/ERK kinase inhibitor (TAK-733 or trametinib); MGMT = O^6 methylguanine DNA methyltransferase; NA = Not applicable; OLA = Olaparib; OS = overall survival; OVT= Oncolytic viral therapy; PAN = Panobinostat; PDX = patient-derived xenograft; PEM = Pembrolizumab; TMZ = Temozolomide; TUB = Tubacin; RT = Radiotherapy; VAN = Vandenatib. * fusion molecule of an alkylator, bendamustine, and vorinostat; & DNA methylation inhibitor; % = proteasome inhibitor.*

References

1. Eyupoglu, I.Y.; Hahnen, E.; Buslei, R.; Siebzehnrubl, F.A.; Savaskan, N.E.; Luders, M.; Trankle, C.; Wick, W.; Weller, M.; Fahlbusch, R.; et al. Suberoylanilide hydroxamic acid (SAHA) has potent anti-glioma properties in vitro, ex vivo and in vivo. *J Neurochem* **2005**, *93*, 992-999, doi:10.1111/j.1471-4159.2005.03098.x.
2. Ugur, H.C.; Ramakrishna, N.; Bello, L.; Menon, L.G.; Kim, S.K.; Black, P.M.; Carroll, R.S. Continuous intracranial administration of suberoylanilide hydroxamic acid (SAHA) inhibits tumor growth in an orthotopic glioma model. *J Neurooncol* **2007**, *83*, 267-275, doi:10.1007/s11060-007-9337-z.
3. Yin, D.; Ong, J.M.; Hu, J.; Desmond, J.C.; Kawamata, N.; Konda, B.M.; Black, K.L.; Koeffler, H.P. Suberoylanilide hydroxamic acid, a histone deacetylase inhibitor: effects on gene expression and growth of glioma cells in vitro and in vivo. *Clin Cancer Res* **2007**, *13*, 1045-1052, doi:10.1158/1078-0432.CCR-06-1261.
4. Jane, E.P.; Premkumar, D.R.; Addo-Yobo, S.O.; Pollack, I.F. Abrogation of mitogen-activated protein kinase and Akt signaling by vandetanib synergistically potentiates histone deacetylase inhibitor-induced apoptosis in human glioma cells. *J Pharmacol Exp Ther* **2009**, *331*, 327-337, doi:10.1124/jpet.109.155705.
5. An, Z.; Gluck, C.B.; Choy, M.L.; Kaufman, L.J. Suberoylanilide hydroxamic acid limits migration and invasion of glioma cells in two and three dimensional culture. *Cancer Lett* **2010**, *292*, 215-227, doi:10.1016/j.canlet.2009.12.006.
6. Orzan, F.; Pellegatta, S.; Poliani, P.L.; Pisati, F.; Caldera, V.; Menghi, F.; Kapetis, D.; Marras, C.; Schiffer, D.; Finocchiaro, G. Enhancer of Zeste 2 (EZH2) is up-regulated in malignant gliomas and in glioma stem-like cells. *Neuropathol Appl Neurobiol* **2011**, *37*, 381-394, doi:10.1111/j.1365-2990.2010.01132.x.
7. Xu, J.; Sampath, D.; Lang, F.F.; Prabhu, S.; Rao, G.; Fuller, G.N.; Liu, Y.; Puduvalli, V.K. Vorinostat modulates cell cycle regulatory proteins in glioma cells and human glioma slice cultures. *J Neurooncol* **2011**, *105*, 241-251, doi:10.1007/s11060-011-0604-7.
8. Singh, M.M.; Manton, C.A.; Bhat, K.P.; Tsai, W.W.; Aldape, K.; Barton, M.C.; Chandra, J. Inhibition of LSD1 sensitizes glioblastoma cells to histone deacetylase inhibitors. *Neuro Oncol* **2011**, *13*, 894-903, doi:10.1093/neuonc/nor049.
9. Chiao, M.T.; Cheng, W.Y.; Yang, Y.C.; Shen, C.C.; Ko, J.L. Suberoylanilide hydroxamic acid (SAHA) causes tumor growth slowdown and triggers autophagy in glioblastoma stem cells. *Autophagy* **2013**, *9*, 1509-1526, doi:10.4161/auto.25664.
10. Premkumar, D.R.; Jane, E.P.; Agostino, N.R.; DiDomenico, J.D.; Pollack, I.F. Bortezomib-induced sensitization of malignant human glioma cells to vorinostat-induced apoptosis depends on reactive oxygen species production, mitochondrial dysfunction, Noxa upregulation, Mcl-1 cleavage, and DNA damage. *Mol Carcinog* **2013**, *52*, 118-133, doi:10.1002/mc.21835.
11. Berghauser Pont, L.M.; Spoor, J.K.; Venkatesan, S.; Swagemakers, S.; Kloezeman, J.J.; Dirven, C.M.; van der Spek, P.J.; Lamfers, M.L.; Leenstra, S. The Bcl-2 inhibitor Obatoclax overcomes resistance to histone deacetylase inhibitors SAHA and LBH589 as radiosensitizers in patient-derived glioblastoma stem-like cells. *Genes Cancer* **2014**, *5*, 445-459, doi:10.18632/genesandcancer.42.
12. Cornago, M.; Garcia-Alberich, C.; Blasco-Angulo, N.; Vall-Llaura, N.; Nager, M.; Herreros, J.; Comella, J.X.; Sanchis, D.; Llovera, M. Histone deacetylase inhibitors promote glioma cell death by G2 checkpoint abrogation leading to mitotic catastrophe. *Cell Death Dis* **2014**, *5*, e1435, doi:10.1038/cddis.2014.412.
13. Barazzuol, L.; Jeynes, J.C.; Merchant, M.J.; Wera, A.C.; Barry, M.A.; Kirkby, K.J.; Suzuki, M. Radiosensitization of glioblastoma cells using a histone deacetylase inhibitor (SAHA) comparing carbon ions with X-rays. *Int J Radiat Biol* **2015**, *91*, 90-98, doi:10.3109/09553002.2014.946111.
14. Pont, L.M.; Naipal, K.; Kloezeman, J.J.; Venkatesan, S.; van den Bent, M.; van Gent, D.C.; Dirven, C.M.; Kanaar, R.; Lamfers, M.L.; Leenstra, S. DNA damage response and anti-apoptotic proteins predict radiosensitization efficacy of HDAC inhibitors SAHA and LBH589 in patient-derived glioblastoma cells. *Cancer Lett* **2015**, *356*, 525-535, doi:10.1016/j.canlet.2014.09.049.
15. Rasmussen, R.D.; Gajjar, M.K.; Jensen, K.E.; Hamerlik, P. Enhanced efficacy of combined HDAC and PARP targeting in glioblastoma. *Mol Oncol* **2016**, *10*, 751-763, doi:10.1016/j.molonc.2015.12.014.
16. Zhang, C.; Yang, C.; Feldman, M.J.; Wang, H.; Pang, Y.; Maggio, D.M.; Zhu, D.; Nesvick, C.L.; Dmitriev, P.; Bullova, P.; et al. Vorinostat suppresses hypoxia signaling by modulating nuclear translocation of hypoxia inducible factor 1 alpha. *Oncotarget* **2017**, *8*, 56110-56125, doi:10.18632/oncotarget.18125.
17. Lohitesh, K.; Saini, H.; Srivastava, A.; Mukherjee, S.; Roy, A.; Chowdhury, R. Autophagy inhibition potentiates SAHA-mediated apoptosis in glioblastoma cells by accumulation of damaged mitochondria. *Oncol Rep* **2018**, *39*, 2787-2796, doi:10.3892/or.2018.6373.
18. Gonçalves, R.M.; Agnes, J.P.; Delgobo, M.; de Souza, P.O.; Thomé, M.P.; Heimfarth, L.; Lenz, G.; Moreira, J.C.F.; Zanotto-Filho, A. Late autophagy inhibitor chloroquine improves efficacy of the histone

deacetylase inhibitor SAHA and temozolomide in gliomas. *Biochem Pharmacol* **2019**, *163*, 440-450, doi:10.1016/j.bcp.2019.03.015.
19. Pastorino, O.; Gentile, M.T.; Mancini, A.; Del Gaudio, N.; Di Costanzo, A.; Bajetto, A.; Franco, P.; Altucci, L.; Florio, T.; Stoppelli, M.P.; et al. Histone Deacetylase Inhibitors Impair Vasculogenic Mimicry from Glioblastoma Cells. *Cancers* **2019**, *11*, 747, doi:10.3390/cancers11060747.
20. Khathayer, F.; Taylor, M.A.; Ray, S.K. Synergism of 4HPR and SAHA increases anti-tumor actions in glioblastoma cells. *Apoptosis* **2020**, *25*, 217-232, doi:10.1007/s10495-020-01590-9.
21. Perez, T.; Bergès, R.; Maccario, H.; Oddoux, S.; Honoré, S. Low concentrations of vorinostat decrease EB1 expression in GBM cells and affect microtubule dynamics, cell survival and migration. *Oncotarget* **2021**, *12*, 304-315, doi:10.18632/oncotarget.27892.
22. Rampazzo, E.; Manfreda, L.; Bresolin, S.; Cani, A.; Mariotto, E.; Bortolozzi, R.; Della Puppa, A.; Viola, G.; Persano, L. Histone Deacetylase Inhibitors Impair Glioblastoma Cell Motility and Proliferation. *Cancers* **2022**, *14*, 1897, doi:10.3390/cancers14081897.
23. Qiu, Y.; Li, Z.; Copland, J.A.; Mehrling, T.; Tun, H.W. Combined alkylation and histone deacetylase inhibition with EDO-S101 has significant therapeutic activity against brain tumors in preclinical models. *Oncotarget* **2018**, *9*, 28155-28164, doi:10.18632/oncotarget.25588.
24. Sawa, H.; Murakami, H.; Kumagai, M.; Nakasato, M.; Yamauchi, S.; Matsuyama, N.; Tamura, Y.; Satone, A.; Ide, W.; Hashimoto, I.; et al. Histone deacetylase inhibitor, FK228, induces apoptosis and suppresses cell proliferation of human glioblastoma cells in vitro and in vivo. *Acta Neuropathol* **2004**, *107*, 523-531, doi:10.1007/s00401-004-0841-3.
25. Nguyen, T.T.T.; Zhang, Y.; Shang, E.; Shu, C.; Quinzii, C.M.; Westhoff, M.A.; Karpel-Massler, G.; Siegelin, M.D. Inhibition of HDAC1/2 Along with TRAP1 Causes Synthetic Lethality in Glioblastoma Model Systems. *Cells* **2020**, *9*, 1661, doi:10.3390/cells9071661.
26. Nguyen, T.T.T.; Shang, E.; Schiffgens, S.; Torrini, C.; Shu, C.; Akman, H.O.; Prabhu, V.V.; Allen, J.E.; Westhoff, M.A.; Karpel-Massler, G.; et al. Induction of Synthetic Lethality by Activation of Mitochondrial ClpP and Inhibition of HDAC1/2 in Glioblastoma. *Clin Cancer Res* **2022**, *28*, 1881-1895, doi:10.1158/1078-0432.ccr-21-2857.
27. Kusaczuk, M.; Kretowski, R.; Stypulkowska, A.; Cechowska-Pasko, M. Molecular and cellular effects of a novel hydroxamate-based HDAC inhibitor - belinostat - in glioblastoma cell lines: a preliminary report. *Invest New Drugs* **2016**, *34*, 552-564, doi:10.1007/s10637-016-0372-5.
28. Gurbani, S.S.; Yoon, Y.; Weinberg, B.D.; Salgado, E.; Press, R.H.; Cordova, J.S.; Ramesh, K.K.; Liang, Z.; Velazquez Vega, J.; Voloschin, A.; et al. Assessing Treatment Response of Glioblastoma to an HDAC Inhibitor Using Whole-Brain Spectroscopic MRI. *Tomography* **2019**, *5*, 53-60, doi:10.18383/j.tom.2018.00031.
29. Yu, C.; Friday, B.B.; Yang, L.; Atadja, P.; Wigle, D.; Sarkaria, J.; Adjei, A.A. Mitochondrial Bax translocation partially mediates synergistic cytotoxicity between histone deacetylase inhibitors and proteasome inhibitors in glioma cells. *Neuro-oncol* **2008**, *10*, 309-319, doi:10.1215/15228517-2007-063.
30. Berghauser Pont, L.M.; Kleijn, A.; Kloezeman, J.J.; van den Bossche, W.; Kaufmann, J.K.; de Vrij, J.; Leenstra, S.; Dirven, C.M.; Lamfers, M.L. The HDAC Inhibitors Scriptaid and LBH589 Combined with the Oncolytic Virus Delta24-RGD Exert Enhanced Anti-Tumor Efficacy in Patient-Derived Glioblastoma Cells. *PLoS One* **2015**, *10*, e0127058, doi:10.1371/journal.pone.0127058.
31. Singleton, W.G.; Collins, A.M.; Bienemann, A.S.; Killick-Cole, C.L.; Haynes, H.R.; Asby, D.J.; Butts, C.P.; Wyatt, M.J.; Barua, N.U.; Gill, S.S. Convection enhanced delivery of panobinostat (LBH589)-loaded pluronic nano-micelles prolongs survival in the F98 rat glioma model. *Int J Nanomedicine* **2017**, *12*, 1385-1399, doi:10.2147/ijn.s125300.
32. Yao, Z.G.; Li, W.H.; Hua, F.; Cheng, H.X.; Zhao, M.Q.; Sun, X.C.; Qin, Y.J.; Li, J.M. LBH589 Inhibits Glioblastoma Growth and Angiogenesis Through Suppression of HIF-1α Expression. *J Neuropathol Exp Neurol* **2017**, *76*, 1000-1007, doi:10.1093/jnen/nlx088.
33. Meng, W.; Wang, B.; Mao, W.; Wang, J.; Zhao, Y.; Li, Q.; Zhang, C.; Tang, Y.; Ma, J. Enhanced efficacy of histone deacetylase inhibitor combined with bromodomain inhibitor in glioblastoma. *J Exp Clin Cancer Res* **2018**, *37*, 241, doi:10.1186/s13046-018-0916-y.
34. Zhang, Y.; Ishida, C.T.; Ishida, W.; Lo, S.L.; Zhao, J.; Shu, C.; Bianchetti, E.; Kleiner, G.; Sanchez-Quintero, M.J.; Quinzii, C.M.; et al. Combined HDAC and Bromodomain Protein Inhibition Reprograms Tumor Cell Metabolism and Elicits Synthetic Lethality in Glioblastoma. *Clin Cancer Res* **2018**, *24*, 3941-3954, doi:10.1158/1078-0432.ccr-18-0260.
35. Meng, W.; Wang, B.; Mao, W.; Wang, J.; Zhao, Y.; Li, Q.; Zhang, C.; Ma, J. Enhanced efficacy of histone deacetylase inhibitor panobinostat combined with dual PI3K/mTOR inhibitor BEZ235 against glioblastoma. *Nagoya J Med Sci* **2019**, *81*, 93-102, doi:10.18999/nagjms.81.1.93.

36. Nguyen, T.T.T.; Zhang, Y.; Shang, E.; Shu, C.; Torrini, C.; Zhao, J.; Bianchetti, E.; Mela, A.; Humala, N.; Mahajan, A.; et al. HDAC inhibitors elicit metabolic reprogramming by targeting super-enhancers in glioblastoma models. *J Clin Invest* **2020**, *130*, 3699-3716, doi:10.1172/jci129049.
37. De La Rosa, J.; Urdiciain, A.; Zazpe, I.; Zelaya, M.V.; Meléndez, B.; Rey, J.A.; Idoate, M.A.; Castresana, J.S. The synergistic effect of DZ-NEP, panobinostat and temozolomide reduces clonogenicity and induces apoptosis in glioblastoma cells. *Int J Oncol* **2020**, *56*, 283-300, doi:10.3892/ijo.2019.4905.
38. De La Rosa, J.; Urdiciain, A.; Zelaya, M.V.; Zazpe, I.; Meléndez, B.; Rey, J.A.; Idoate, M.A.; Castresana, J.S. APR-246 combined with 3-deazaneplanocin A, panobinostat or temozolomide reduces clonogenicity and induces apoptosis in glioblastoma cells. *Int J Oncol* **2021**, *58*, 312-330, doi:10.3892/ijo.2021.5177.
39. Franco-Molina, M.A.; Santana-Krímskaya, S.E.; Madrigal-de-León, L.M.; Coronado-Cerda, E.E.; Zárate-Triviño, D.G.; Hernández-Martínez, S.P.; García-Coronado, P.L.; Rodríguez-Padilla, C. Evaluation of the cytotoxic and immunogenic potential of temozolamide, panobinostat, and Lophophora williamsii extract against C6 glioma cells. *Excli j* **2021**, *20*, 614-624, doi:10.17179/excli2020-3181.
40. Pratap, U.P.; Sareddy, G.R.; Liu, Z.; Venkata, P.P.; Liu, J.; Tang, W.; Altwegg, K.A.; Ebrahimi, B.; Li, X.; Tekmal, R.R.; et al. Histone deacetylase inhibitors enhance estrogen receptor beta expression and augment agonist-mediated tumor suppression in glioblastoma. *Neurooncol Adv* **2021**, *3*, vdab099, doi:10.1093/noajnl/vdab099.
41. Bacon, C.L.; O'Driscoll, E.; Regan, C.M. Valproic acid suppresses G1 phase-dependent sialylation of a 65 kDa glycoprotein in the C6glioma cell cycle. *Int J Dev Neurosci* **1998**, *15*, 777-784, doi:10.1016/s0736-5748(97)00019-1.
42. Knupfer, M.M.; Hernaiz-Driever, P.; Poppenborg, H.; Wolff, J.E.; Cinatl, J. Valproic acid inhibits proliferation and changes expression of CD44 and CD56 of malignant glioma cells in vitro. *Anticancer Res* **1998**, *18*, 3585-3589.
43. Camphausen, K.; Cerna, D.; Scott, T.; Sproull, M.; Burgan, W.E.; Cerra, M.A.; Fine, H.; Tofilon, P.J. Enhancement of in vitro and in vivo tumor cell radiosensitivity by valproic acid. *Int J Cancer* **2005**, *114*, 380-386, doi:10.1002/ijc.20774.
44. Chavez-Blanco, A.; Perez-Plasencia, C.; Perez-Cardenas, E.; Carrasco-Legleu, C.; Rangel-Lopez, E.; Segura-Pacheco, B.; Taja-Chayeb, L.; Trejo-Becerril, C.; Gonzalez-Fierro, A.; Candelaria, M.; et al. Antineoplastic effects of the DNA methylation inhibitor hydralazine and the histone deacetylase inhibitor valproic acid in cancer cell lines. *Cancer cell international* **2006**, *6*, 2, doi:10.1186/1475-2867-6-2.
45. Das, C.M.; Aguilera, D.; Vasquez, H.; Prasad, P.; Zhang, M.; Wolff, J.E.; Gopalakrishnan, V. Valproic acid induces p21 and topoisomerase-II (alpha/beta) expression and synergistically enhances etoposide cytotoxicity in human glioblastoma cell lines. *J Neurooncol* **2007**, *85*, 159-170, doi:10.1007/s11060-007-9402-7.
46. Papi, A.; Ferreri, A.M.; Rocchi, P.; Guerra, F.; Orlandi, M. Epigenetic Modifiers as Anticancer Drugs: Effectiveness of Valproic Acid in Neural Crest-derived Tumor Cells. *Anticancer Res* **2010**, *30*, 535-540.
47. Alvarez, A.A.; Field, M.; Bushnev, S.; Longo, M.S.; Sugaya, K. The effects of histone deacetylase inhibitors on glioblastoma-derived stem cells. *J Mol Neurosci* **2015**, *55*, 7-20, doi:10.1007/s12031-014-0329-0.
48. Zhang, C.; Liu, S.; Yuan, X.; Hu, Z.; Li, H.; Wu, M.; Yuan, J.; Zhao, Z.; Su, J.; Wang, X.; et al. Valproic Acid Promotes Human Glioma U87 Cells Apoptosis and Inhibits Glycogen Synthase Kinase-3β Through ERK/Akt Signaling. *Cell Physiol Biochem* **2016**, *39*, 2173-2185, doi:10.1159/000447912.
49. Chang, Y.L.; Huang, L.C.; Chen, Y.C.; Wang, Y.W.; Hueng, D.Y.; Huang, S.M. The synergistic effects of valproic acid and fluvastatin on apoptosis induction in glioblastoma multiforme cell lines. *Int J Biochem Cell Biol* **2017**, *92*, 155-163, doi:10.1016/j.biocel.2017.10.003.
50. Tseng, J.H.; Chen, C.Y.; Chen, P.C.; Hsiao, S.H.; Fan, C.C.; Liang, Y.C.; Chen, C.P. Valproic acid inhibits glioblastoma multiforme cell growth via paraoxonase 2 expression. *Oncotarget* **2017**, *8*, 14666-14679, doi:10.18632/oncotarget.14716.
51. Garcia, C.G.; Kahn, S.A.; Geraldo, L.H.M.; Romano, I.; Domith, I.; Silva, D.; Dos Santos Assunção, F.; Ferreira, M.J.; Portugal, C.C.; de Souza, J.M.; et al. Combination Therapy with Sulfasalazine and Valproic Acid Promotes Human Glioblastoma Cell Death Through Imbalance of the Intracellular Oxidative Response. *Mol Neurobiol* **2018**, *55*, 6816-6833, doi:10.1007/s12035-018-0895-1.
52. Riva, G.; Cilibrasi, C.; Bazzoni, R.; Cadamuro, M.; Negroni, C.; Butta, V.; Strazzabosco, M.; Dalprà, L.; Lavitrano, M.; Bentivegna, A. Valproic Acid Inhibits Proliferation and Reduces Invasiveness in Glioma Stem Cells Through Wnt/β Catenin Signalling Activation. *Genes* **2018**, *9*, 522, doi:10.3390/genes9110522.
53. Chen, J.C.; Lee, I.N.; Huang, C.; Wu, Y.P.; Chung, C.Y.; Lee, M.H.; Lin, M.H.; Yang, J.T. Valproic acid-induced amphiregulin secretion confers resistance to temozolomide treatment in human glioma cells. *BMC Cancer* **2019**, *19*, 756, doi:10.1186/s12885-019-5843-6.

54. Berendsen, S.; Frijlink, E.; Kroonen, J.; Spliet, W.G.M.; van Hecke, W.; Seute, T.; Snijders, T.J.; Robe, P.A. Effects of valproic acid on histone deacetylase inhibition in vitro and in glioblastoma patient samples. *Neurooncol Adv* **2019**, *1*, vdz025, doi:10.1093/noajnl/vdz025.
55. Sanaei, M.; Kavoosi, F. The effect of valproic acid on intrinsic, extrinsic, and JAK/STAT pathways in neuroblastoma and glioblastoma cell lines. *Res Pharm Sci* **2022**, *17*, 392-409, doi:10.4103/1735-5362.350240.
56. Tarasenko, N.; Chekroun-Setti, H.; Nudelman, A.; Rephaeli, A. Comparison of the anticancer properties of a novel valproic acid prodrug to leading histone deacetylase inhibitors. *J Cell Biochem* **2018**, *119*, 3417-3428, doi:10.1002/jcb.26512.
57. Sawa, H.; Murakami, H.; Ohshima, Y.; Sugino, T.; Nakajyo, T.; Kisanuki, T.; Tamura, Y.; Satone, A.; Ide, W.; Hashimoto, I.; et al. Histone deacetylase inhibitors such as sodium butyrate and trichostatin A induce apoptosis through an increase of the bcl-2-related protein Bad. *Brain Tumor Pathol* **2001**, *18*, 109-114, doi:10.1007/bf02479423.
58. Wetzel, M.; Premkumar, D.R.; Arnold, B.; Pollack, I.F. Effect of trichostatin A, a histone deacetylase inhibitor, on glioma proliferation in vitro by inducing cell cycle arrest and apoptosis. *J Neurosurg* **2005**, *103*, 549-556, doi:10.3171/ped.2005.103.6.0549.
59. Egler, V.; Korur, S.; Failly, M.; Boulay, J.L.; Imber, R.; Lino, M.M.; Merlo, A. Histone deacetylase inhibition and blockade of the glycolytic pathway synergistically induce glioblastoma cell death. *Clin Cancer Res* **2008**, *14*, 3132-3140, doi:10.1158/1078-0432.CCR-07-4182.
60. Svechnikova, I.; Almqvist, P.M.; Ekstrom, T.J. HDAC inhibitors effectively induce cell type-specific differentiation in human glioblastoma cell lines of different origin. *Int J Oncol* **2008**, *32*, 821-827, doi:10.3892/ijo.32.4.821.
61. Gao, J.; Chen, T.; Liu, J.; Liu, W.; Hu, G.; Guo, X.; Yin, B.; Gong, Y.; Zhao, J.; Qiang, B.; et al. Loss of NECL1, a novel tumor suppressor, can be restored in glioma by HDAC inhibitor-Trichostatin A through Sp1 binding site. *Glia* **2009**, *57*, 989-999, doi:10.1002/glia.20823.
62. Foltz, G.; Yoon, J.G.; Lee, H.; Ma, L.; Tian, Q.; Hood, L.; Madan, A. Epigenetic regulation of wnt pathway antagonists in human glioblastoma multiforme. *Genes Cancer* **2010**, *1*, 81-90, doi:10.1177/1947601909356103.
63. Hsu, Y.F.; Sheu, J.R.; Hsiao, G.; Lin, C.H.; Chang, T.H.; Chiu, P.T.; Wang, C.Y.; Hsu, M.J. p53 in trichostatin A induced C6 glioma cell death. *Biochim Biophys Acta* **2011**, *1810*, 504-513, doi:10.1016/j.bbagen.2011.02.006.
64. Horing, E.; Podlech, O.; Silkenstedt, B.; Rota, I.A.; Adamopoulou, E.; Naumann, U. The histone deacetylase inhibitor trichostatin a promotes apoptosis and antitumor immunity in glioblastoma cells. *Anticancer Res* **2013**, *33*, 1351-1360.
65. Sassi Fde, A.; Caesar, L.; Jaeger, M.; Nor, C.; Abujamra, A.L.; Schwartsmann, G.; de Farias, C.B.; Brunetto, A.L.; Lopez, P.L.; Roesler, R. Inhibitory activities of trichostatin a in U87 glioblastoma cells and tumorsphere-derived cells. *J Mol Neurosci* **2014**, *54*, 27-40, doi:10.1007/s12031-014-0241-7.
66. Staberg, M.; Michaelsen, S.R.; Rasmussen, R.D.; Villingshoj, M.; Poulsen, H.S.; Hamerlik, P. Inhibition of histone deacetylases sensitizes glioblastoma cells to lomustine. *Cell Oncol* **2017**, *40*, 21-32, doi:10.1007/s13402-016-0301-9.
67. Alexanian, A.R.; Brannon, A. Unique combinations of epigenetic modifiers synergistically impair the viability of the U87 glioblastoma cell line while exhibiting minor or moderate effects on normal stem cell growth. *Med Oncol* **2022**, *39*, 86, doi:10.1007/s12032-022-01683-2.
68. Camphausen, K.; Burgan, W.; Cerra, M.; Oswald, K.A.; Trepel, J.B.; Lee, M.J.; Tofilon, P.J. Enhanced radiation-induced cell killing and prolongation of gammaH2AX foci expression by the histone deacetylase inhibitor MS-275. *Cancer Res* **2004**, *64*, 316-321, doi:10.1158/0008-5472.can-03-2630.
69. Eyupoglu, I.Y.; Hahnen, E.; Trankle, C.; Savaskan, N.E.; Siebzehnrubl, F.A.; Buslei, R.; Lemke, D.; Wick, W.; Fahlbusch, R.; Blumcke, I. Experimental therapy of malignant gliomas using the inhibitor of histone deacetylase MS-275. *Mol Cancer Ther* **2006**, *5*, 1248-1255, doi:10.1158/1535-7163.MCT-05-0533.
70. Sun, P.; Xia, S.; Lal, B.; Eberhart, C.G.; Quinones-Hinojosa, A.; Maciaczyk, J.; Matsui, W.; Dimeco, F.; Piccirillo, S.M.; Vescovi, A.L.; et al. DNER, an epigenetically modulated gene, regulates glioblastoma-derived neurosphere cell differentiation and tumor propagation. *Stem cells* **2009**, *27*, 1473-1486, doi:10.1002/stem.89.
71. Bangert, A.; Hacker, S.; Cristofanon, S.; Debatin, K.M.; Fulda, S. Chemosensitization of glioblastoma cells by the histone deacetylase inhibitor MS275. *Anticancer Drugs* **2011**, *22*, 494-499, doi:10.1097/CAD.0b013e32834631e0.
72. Essien, E.I.; Hofer, T.P.; Atkinson, M.J.; Anastasov, N. Combining HDAC and MEK Inhibitors with Radiation against Glioblastoma-Derived Spheres. *Cells* **2022**, *11*, 775, doi:10.3390/cells11050775.

73. Buyandelger, B.; Bar, E.E.; Hung, K.S.; Chen, R.M.; Chiang, Y.H.; Liou, J.P.; Huang, H.M.; Wang, J.Y. Histone deacetylase inhibitor MPT0B291 suppresses Glioma Growth in vitro and in vivo partially through acetylation of p53. *Int J Biol Sci* **2020**, *16*, 3184-3199, doi:10.7150/ijbs.45505.
74. Yang, W.B.; Wu, A.C.; Hsu, T.I.; Liou, J.P.; Lo, W.L.; Chang, K.Y.; Chen, P.Y.; Kikkawa, U.; Yang, S.T.; Kao, T.J.; et al. Histone deacetylase 6 acts upstream of DNA damage response activation to support the survival of glioblastoma cells. *Cell Death Dis* **2021**, *12*, 884, doi:10.1038/s41419-021-04182-w.
75. Carol, H.; Gorlick, R.; Kolb, E.A.; Morton, C.L.; Manesh, D.M.; Keir, S.T.; Reynolds, C.P.; Kang, M.H.; Maris, J.M.; Wozniak, A.; et al. Initial testing (stage 1) of the histone deacetylase inhibitor, quisinostat (JNJ-26481585), by the Pediatric Preclinical Testing Program. *Pediatr Blood Cancer* **2014**, *61*, 245-252, doi:10.1002/pbc.24724.
76. Bouché, M.; Dong, Y.C.; Sheikh, S.; Taing, K.; Saxena, D.; Hsu, J.C.; Chen, M.H.; Salinas, R.D.; Song, H.; Burdick, J.A.; et al. Novel Treatment for Glioblastoma Delivered by a Radiation Responsive and Radiopaque Hydrogel. *ACS Biomater Sci Eng* **2021**, *7*, 3209-3220, doi:10.1021/acsbiomaterials.1c00385.
77. Choi, S.A.; Kwak, P.A.; Park, C.K.; Wang, K.C.; Phi, J.H.; Lee, J.Y.; Lee, C.S.; Lee, J.H.; Kim, S.K. A novel histone deacetylase inhibitor, CKD5, has potent anti-cancer effects in glioblastoma. *Oncotarget* **2017**, *8*, 9123-9133, doi:10.18632/oncotarget.13265.
78. Zhang, I.; Beus, M.; Stochaj, U.; Le, P.U.; Zorc, B.; Rajic, Z.; Petrecca, K.; Maysinger, D. Inhibition of glioblastoma cell proliferation, invasion, and mechanism of action of a novel hydroxamic acid hybrid molecule. *Cell death discov* **2018**, *4*, 41, doi:10.1038/s41420-018-0103-0.
79. Zhang, W.; Lv, S.; Liu, J.; Zang, Z.; Yin, J.; An, N.; Yang, H.; Song, Y. PCI-24781 down-regulates EZH2 expression and then promotes glioma apoptosis by suppressing the PIK3K/Akt/mTOR pathway. *Genet Mol Biol* **2014**, *37*, 716-724, doi:10.1590/S1415-47572014005000011.
80. Vengoji, R.; Atri, P.; Macha, M.A.; Seshacharyulu, P.; Perumal, N.; Mallya, K.; Liu, Y.; Smith, L.M.; Rachagani, S.; Mahapatra, S.; et al. Differential gene expression-based connectivity mapping identified novel drug candidate and improved Temozolomide efficacy for Glioblastoma. *J Exp Clin Cancer Res* **2021**, *40*, 335, doi:10.1186/s13046-021-02135-x.
81. Asklund, T.; Appelskog, I.B.; Ammerpohl, O.; Ekstrom, T.J.; Almqvist, P.M. Histone deacetylase inhibitor 4-phenylbutyrate modulates glial fibrillary acidic protein and connexin 43 expression, and enhances gap-junction communication, in human glioblastoma cells. *Eur J Cancer* **2004**, *40*, 1073-1081, doi:10.1016/j.ejca.2003.11.034.
82. Marino, A.M.; Sofiadis, A.; Baryawno, N.; Johnsen, J.I.; Larsson, C.; Vukojevic, V.; Ekstrom, T.J. Enhanced effects by 4-phenylbutyrate in combination with RTK inhibitors on proliferation in brain tumor cell models. *Biochem Biophys Res Commun* **2011**, *411*, 208-212, doi:10.1016/j.bbrc.2011.06.141.
83. Kusaczuk, M.; Krętowski, R.; Bartoszewicz, M.; Cechowska-Pasko, M. Phenylbutyrate-a pan-HDAC inhibitor-suppresses proliferation of glioblastoma LN-229 cell line. *Tumour Biol* **2016**, *37*, 931-942, doi:10.1007/s13277-015-3781-8.
84. Engelhard, H.H.; Duncan, H.A.; Kim, S.; Criswell, P.S.; Van Eldik, L. Therapeutic effects of sodium butyrate on glioma cells in vitro and in the rat C6 glioma model. *Neurosurgery* **2001**, *48*, 616-624, doi:10.1097/00006123-200103000-00035.
85. Nakagawa, H.; Sasagawa, S.; Itoh, K. Sodium butyrate induces senescence and inhibits the invasiveness of glioblastoma cells. *Oncol Lett* **2018**, *15*, 1495-1502, doi:10.3892/ol.2017.7518.
86. Taylor, M.A.; Khathayer, F.; Ray, S.K. Quercetin and Sodium Butyrate Synergistically Increase Apoptosis in Rat C6 and Human T98G Glioblastoma Cells Through Inhibition of Autophagy. *Neurochem Res* **2019**, *44*, 1715-1725, doi:10.1007/s11064-019-02802-8.
87. Majchrzak-Celińska, A.; Kleszcz, R.; Stasiłowicz-Krzemień, A.; Cielecka-Piontek, J. Sodium Butyrate Enhances Curcuminoids Permeability through the Blood-Brain Barrier, Restores Wnt/β-Catenin Pathway Antagonists Gene Expression and Reduces the Viability of Glioblastoma Cells. *Int J Mol Sci* **2021**, *22*, 11285, doi:10.3390/ijms222011285.
88. Pająk, B.; Siwiak-Niedbalska, E.; Jaśkiewicz, A.; Sołtyka, M.; Zieliński, R.; Domoradzki, T.; Fokt, I.; Skóra, S.; Priebe, W. Synergistic Anticancer Effect of Glycolysis and Histone Deacetylases Inhibitors in a Glioblastoma Model. *Biomedicines* **2021**, *9*, 1749, doi:10.3390/biomedicines9121749.
89. Zhang, G.; Gan, Y.H. Synergistic antitumor effects of the combined treatment with an HDAC6 inhibitor and a COX-2 inhibitor through activation of PTEN. *Oncol Rep* **2017**, *38*, 2657-2666, doi:10.3892/or.2017.5981.
90. Kim, G.W.; Lee, D.H.; Yeon, S.K.; Jeon, Y.H.; Yoo, J.; Lee, S.W.; Kwon, S.H. Temozolomide-resistant Glioblastoma Depends on HDAC6 Activity Through Regulation of DNA Mismatch Repair. *Anticancer Res* **2019**, *39*, 6731-6741, doi:10.21873/anticanres.13888.

91. Urdiciain, A.; Erausquin, E.; Meléndez, B.; Rey, J.A.; Idoate, M.A.; Castresana, J.S. Tubastatin A, an inhibitor of HDAC6, enhances temozolomide-induced apoptosis and reverses the malignant phenotype of glioblastoma cells. *Int J Oncol* **2019**, *54*, 1797-1808, doi:10.3892/ijo.2019.4739.
92. Auzmendi-Iriarte, J.; Saenz-Antoñanzas, A.; Mikelez-Alonso, I.; Carrasco-Garcia, E.; Tellaetxe-Abete, M.; Lawrie, C.H.; Sampron, N.; Cortajarena, A.L.; Matheu, A. Characterization of a new small-molecule inhibitor of HDAC6 in glioblastoma. *Cell Death Discov* **2020**, *11*, 417, doi:10.1038/s41419-020-2586-x.
93. Yin, C.; Li, P. Growth Suppression of Glioma Cells Using HDAC6 Inhibitor, Tubacin. *Open Med* **2018**, *13*, 221-226, doi:10.1515/med-2018-0034.
94. Li, S.; Liu, X.; Chen, X.; Zhang, L.; Wang, X. Histone deacetylase 6 promotes growth of glioblastoma through inhibition of SMAD2 signaling. *Tumour Biol* **2015**, *36*, 9661-9665, doi:10.1007/s13277-015-3747-x.
95. Huang, Z.; Xia, Y.; Hu, K.; Zeng, S.; Wu, L.; Liu, S.; Zhi, C.; Lai, M.; Chen, D.; Xie, L.; et al. Histone deacetylase 6 promotes growth of glioblastoma through the MKK7/JNK/c-Jun signaling pathway. *J Neurochem* **2020**, *152*, 221-234, doi:10.1111/jnc.14849.
96. Liffers, K.; Kolbe, K.; Westphal, M.; Lamszus, K.; Schulte, A. Histone Deacetylase Inhibitors Resensitize EGFR/EGFRvIII-Overexpressing, Erlotinib-Resistant Glioblastoma Cells to Tyrosine Kinase Inhibition. *Target Oncol* **2016**, *11*, 29-40, doi:10.1007/s11523-015-0372-y.
97. Was, H.; Krol, S.K.; Rotili, D.; Mai, A.; Wojtas, B.; Kaminska, B.; Maleszewska, M. Histone deacetylase inhibitors exert anti-tumor effects on human adherent and stem-like glioma cells. *Clin Epigenetics* **2019**, *11*, 11, doi:10.1186/s13148-018-0598-5.
98. Sharma, V.; Koul, N.; Joseph, C.; Dixit, D.; Ghosh, S.; Sen, E. HDAC inhibitor, scriptaid, induces glioma cell apoptosis through JNK activation and inhibits telomerase activity. *J Cell Mol Med* **2010**, *14*, 2151-2161, doi:10.1111/j.1582-4934.2009.00844.x.
99. Balasubramanian, S.; Ramos, J.; Luo, W.; Sirisawad, M.; Verner, E.; Buggy, J.J. A novel histone deacetylase 8 (HDAC8)-specific inhibitor PCI-34051 induces apoptosis in T-cell lymphomas. *Leukemia* **2008**, *22*, 1026-1034, doi:10.1038/leu.2008.9.
100. Marampon, F.; Leoni, F.; Mancini, A.; Pietrantoni, I.; Codenotti, S.; Ferella, L.; Megiorni, F.; Porro, G.; Galbiati, E.; Pozzi, P.; et al. Histone deacetylase inhibitor ITF2357 (givinostat) reverts transformed phenotype and counteracts stemness in in vitro and in vivo models of human glioblastoma. *J Cancer Res Clin Oncol* **2019**, *145*, 393-409, doi:10.1007/s00432-018-2800-8.
101. Angeletti, F.; Fossati, G.; Pattarozzi, A.; Wurth, R.; Solari, A.; Daga, A.; Masiello, I.; Barbieri, F.; Florio, T.; Comincini, S. Inhibition of the Autophagy Pathway Synergistically Potentiates the Cytotoxic Activity of Givinostat (ITF2357) on Human Glioblastoma Cancer Stem Cells. *Front Mol Neurosci* **2016**, *9*, 107, doi:10.3389/fnmol.2016.00107.
102. Taiarol, L.; Bigogno, C.; Sesana, S.; Kravicz, M.; Viale, F.; Pozzi, E.; Monza, L.; Carozzi, V.A.; Meregalli, C.; Valtorta, S.; et al. Givinostat-Liposomes: Anti-Tumor Effect on 2D and 3D Glioblastoma Models and Pharmacokinetics. *Cancers* **2022**, *14*, 2978, doi:10.3390/cancers14122978.
103. Li, Z.Y.; Li, Q.Z.; Chen, L.; Chen, B.D.; Wang, B.; Zhang, X.J.; Li, W.P. Histone Deacetylase Inhibitor RGFP109 Overcomes Temozolomide Resistance by Blocking NF-κB-Dependent Transcription in Glioblastoma Cell Lines. *Neurochem Res* **2016**, *41*, 3192-3205, doi:10.1007/s11064-016-2043-5.
